# Supplementary material for: Empirical methods for controlling false positives and estimating confidence in ChIP-Seq peaks
Source: BMC Bioinformatics. 2008 Dec 5;9:523. doi: 10.1186/1471-2105-9-523 (PMC2628906; doi:10.1186/1471-2105-9-523)
Supplement: Additional File 1 — A variety of html documents from the USeq web site detailing the available applications, their best usage, output file type descriptions, command line menus, etc. [file 1471-2105-9-523-S1.zip › USeqUserGuides/JavaDoc.zip!/JavaDoc/util/bio/wrappers/package-use.html]

Uses of Package util.bio.wrappers


|  |  |  |
| --- | --- | --- |
| **Overview** \| **Package** \| Class \| **Use** \| **Tree** \| **Deprecated** \| **Index** \| **Help** | |  |
| PREV   NEXT | **FRAMES**    **NO FRAMES**     **All Classes** |  |


---


## **Uses of Package util.bio.wrappers**

| Packages that use util.bio.wrappers | |
| --- | --- |
| **bioroot** |  |
| **util.bio.wrappers** | Wrappers for other bio applications. |

Classes in util.bio.wrappers used by bioroot

****BL2SeqHit****
  
          BL2Seq result container.

****BlastResult****
  
          Information related to a blast results.

Classes in util.bio.wrappers used by util.bio.wrappers

****BL2SeqHit****
  
          BL2Seq result container.

****BlastResult****
  
          Information related to a blast results.

****Primer3Pick****
  
          Container for sequence used in picking primers with Primer3.

---


|  |  |  |
| --- | --- | --- |
| **Overview** \| **Package** \| Class \| **Use** \| **Tree** \| **Deprecated** \| **Index** \| **Help** | |  |
| PREV   NEXT | **FRAMES**    **NO FRAMES**     **All Classes** |  |


---
